# Reply to a Slanderous Attack, Published in the Chicago Medical Journal, May Number

**Published:** 1862-07

**Authors:** R. Roskoten

**Affiliations:** Brigade-Surgeon; St. Charles Hotel, Cairo


### Army Correspondence.

### PITTSBURG LANDING.

### REPLY TO A SLANDEROUS ATTACK, PUBLISHED IN THE CHICAGO MEDICAL JOURNAL, MAY NUMBER.

When the present war broke out, and forces were concentrated in numbers never before witnessed on this continent, serious apprehensions were felt for the outbreak of diseases, contagious as well as those so common with recruits in camps. Men of the highest moral and professional standing, therefore, united, and gave the first impulse to the organization of the Sanitary Commission.

Their agents visited the camps, travelled with unabated zeal and ardor over large territories, giving advice to the commanders, and to such medical officers as were not familiar with military hygiene and practice. Their pamphlets on camp policy, on the most frequent and important diseases, on operations, etc., were spread all over the land, and no doubt they accomplished a vast deal of good.

Important improvements in the medical department were adopted on their recommendation, and the establishment of "soldiers' homes" reflects great credit upon them.

Every army officer in the West must have made the acquaintance of Dr. Douglas, the man with an iron constitution and a heart burning with pure benevolence; or of Drs. Warriner and Aigner, highly qualified personages, also inspired by the purest motives.

In short, the Central Sanitary Commission proved itself very useful, and deserves the more credit for it, as in their half-official capacity their mission was a very delicate one. If in any instances they transcended their authority, they were rare and exceptional.

The liberality and benevolence of the American people manifested itself in a manner, and in a degree never before exhibited by any other nation. Articles, not provided for by the army regulations, poured in in such quantities that large warehouses were required for their storage. A noble emulation between the loyal States, resulting in the amplest provision for all the wants of the soldiers. The Sanitary Commission was properly entrusted with the distribution of these gifts. The tree, thus grown up, spread its branches soon over most of the cities.

But the people in selecting their agents were not always very fortunate. We may easily bring them under three distinct classes:—

I. True men, patriotic, devoted with their whole soul to the noble cause they represent.

II. Those who go on an errand only for curiosity sake.

A battle field, after the smoke of the cannon has passed away, affords an exciting and interesting view. You can see these men marching over the field, gathering "trophies," and returning loaded with limbs, and other trash.—Some few of the private physicians may be added to this class, who only come "to amputate." The first and almost stereotyped question, "Is there any operations to be performed," will often be answered in the negative by the surgeon; and if he, insulted by their too often assumed high airs, responds in a manner like this:—"If there is to be one, I shall send my own invitations;" they will retire in double quick.

Finally, there is a set of men, whose only object is to make capital out of it.—They claim the privilege of insulting every army surgeoh who does not bow in devoutest submission to their pretended authority; and I simply ask, who could submit, without being an imbecile, morally or intellectually?

They form a kind of a mutual insurance company, writing occasionally complimentary and glorification letters for each other, as well as for themselves.

To increase the brilliancy of their light, they use the lamp-black very freely for blackguarding.

In their great hurry to have their glory published, they are not always very scrupulous about the correctness of their statements.

Fortunately, their number is not large; but as the members of the first class are generally very modest and kind, devoting all of their time simply to their benevolent objects, and finding no leisure to write hysterical articles for newspapers, their services are mostly overlooked by the outsider, and altogether overshadowed by the conspicuous position their opponents take possession of. These men are the cause of the Sanitary Commission becoming very unpopular among the army officers.

Everybody knows that the distribution of gifts is far more pleasant than the collection of the same; but the art to make presents acceptable is only taught by the most tender feelings of the heart. I always turned away in utter disgust when, to every article given to the poor soldiers, the remark was added: "This comes from the Sanitary Commission!" Why not say, if anything at all, "This comes from the loyal people of the North, from your friends; use it, and be happy!"

It may be mentioned here, that on the 18th of April, a letter from Rev. C. was published in the *Chicago Tribune*, giving an account of the operations of the Chicago Sanitary Commission. The author made some erroneous statements, which could hardly be otherwise in the pressure of a very limited time. Prejudiced, as it seems, in favor of that Commission, he bestowed more credit upon the members than they deserved; while the army surgeons, as a class, were charged with carelessness. At home, on sick leave, I felt myself in duty bound to a reply, correcting some errors, and defending the honor of the former, concluding thus: "It is laudable when citizens and private physicians leave their comfortable homes, or their practice, for a few days, to relieve the unfortunate victims of this war; but their praise must not be heralded at the cost of the army surgeons. And while, through the exertion the writing of this letter causes me, the hemorrhage of my lungs commences anew, I shall never, even when far away, cease to defend the honor of these useful men—men who live with the soldiers, march, suffer, and even die, with them."

This communication, dated April 19, was sent to the *Chicago Tribune* for publication; but my just and reasonable demand was not granted. I then asked for the return of my manuscript, but it was withheld, though postage stamps were sent. It found its way afterwards to the public through the liberality of the *Missouri Republican*, where it was published on the 2d of May. It was never responded to.

But in the *Chicago Medical Journal* appeared an article, headed "Pittsburg Landing," and signed "R." containing the grossest attack upon myself and a colleague of mine. Its spirit and language will contribute a rare sample to the *Ethics* of our time, and it ought to be preserved at Barnum's.

The author is *Dr. Rea, of Chicago.* In his threefold dignity as lecturer in Rush Medical College, as contributor to the *Chicago Medical Journal*, (the organ of said college,) and as *Sanitarian*, he ascends the Olymp and hurls his thunderbolts in a terrible multitude upon us poor mortals. I had the honor to receive a full shower of them, cold, however, and harmless they were.

The gentleman seems to be unaware that this kind of pyrotechnics are "played out," and that such artists burn their fingers occasionally with the same rockets they designed for others.

Living in a skeptical century, rational people are accustomed to respect in science nothing short of *brains;* the mere prestige of a professorship, even in Rush Medical College, belongs to those illusions that were.

I deny the ability of Dr. Rea to judge about me, at all events, if he had even an opportunity for it, which in fact he had not. His very first statement is utterly false. He says, "The boat (Hiawatha) was under charge of Dr. Roskoten." I had no more charge of her than of the St. Charles Hotel, where I am now detained from joining the army, in consequence of the injuries received at Shiloh.

Here is a synopsis of the incidents before and after the arrival of the Chicago Sanitary Commission:—

I arrived at Pittsburg Landing on the 4th of April.

Sunday morning, April 6th, the battle commenced.

Dr. Hewit, the senior medical officer, was acting medical director. I repaired to the medical purveyor's office, to ascertain the condition of the medical supplies.

Answer. Hardly anything; not a grain of opium or morphia.

I then rode to the battle field. About noon, I was struck by a spent piece of shell, in my right side, and at the same time, my horse was killed under me, by a cannon ball. He fell upon me with great force, and it was sometime before, by assistance, I could be extricated. Carried to an ambulance; I was conveyed to *my quarters* on board the Hiawatha.

She contained the commissary stores; the staff of our division (2d) were also quartered there; and we *paid for our board.*

During the day of the battle, a number of wounded officers were brought on board; and before evening some 70 or 80 were quartered there. Though suffering with pain, with profuse hemorrhage from my lungs, and quite lame, requiring surgical attendance myself, I could not resist my desire to relieve the sufferings of my comrades. No surgeon being present, I attended to all of the wounded on board, and I had the satisfaction, of enjoying the gratitude of all of them, notwithstanding the *kind* remark of the very learned professor, "when it was found out that he was incapable of taking care of himself even." True, I did not take proper care of myself in view of the suffering of others, and herein is the cause of my own protracted suffering, and of the probability of never recovering again.

During the full six days, there occurred only one case of death on board the Hiawatha. A minnie ball had caused fracture of the femur, and mortification was present when the man came on board.

The sixth day of hard toil had nearly passed away, when the Chicago Sanitary Commission arrived. They came with a large force of surgeons and some nurses.

The next day, when I was preparing to leave the boat, in order to change my quarters, and to find the rest I needed so much, Dr. Hewit came on board, and stated that this boat was designed to carry wounded men to Mound City Hospital. He *asked me to take charge of her*, as I had to go on sick leave the same way, and as he would rather see the boat in charge of an army surgeon.

Bothered by the new-comers, for medicines and hospital stores, and knowing there were none to be had, I made out my requisitions however, first in the proper form, then on a rag of paper. I never saw my papers again, nor medicines, with the exception of a few trifling articles.

Pressed from a certain side to make requisition for an unreasonable amount of rations, I refused my signature, and asked first for a written order to prove my authority. Then I drew the rations in quantities as I thought proper. The learned Dr. seems to know very little of military matters, and may call it "red tape" if he chooses. So much for the hospital stores and rations.

*By a mere act of courtesy, I transferred the directory of the medical department to Dr. Rea.* I preferred then to be as little official as possible.

Now, the very learned professor says, "The only operation I saw him perform, was trying to squeeze pus from an edematous eyelid." Indeed, Sir.

In passing through the cabin, my attention was attracted by the continuous screaming of a patient. A small ball had entered near the left eyebrow. The eyelid was edematous, the tension enormous. He was crazy with pain, and utterly refused an examination. Stimulated by the desire to relieve this poor fellow somewhat, I scarified that eyelid, and tried to promote the exit of serum and blood.

But as the learned professor seems to have a desire to challenge me in the professional arena, I will lift his glove. In doing so, I may be permitted to publish some of the antediluvial surgery practiced on board the Hiawatha. In passing through the wards to my state-room, some of my former patients complained bitterly of pain. Inquiring for the cause and the treatment, they stated that the cold water dressing had been used. This was done on the 7th day after the injuries, when suppuration and sloughing was fully established.

A private, a German, came on board a few hours before the Commission arrived. Gun-shot, fracture above the knee. Having used all my own splints, I requested Dr. Gillette to attend to this important case. He kindly applied the splint. In passing by the next day, Dr. G. stopped me, the splint was removed. With an almost shy reluctance, he stated to me "That some of the physicians had declared there was no fracture." It was a plain case, every layman, with common sense, could see it at once, and crepitation was audible from a distance. I referred him to Dr. Rea, and a splint was applied again. Comments unnecessary.

An officer, shot in the back, his lower extremities paralysed, also the sphincter ani and bladder. The ball being inextricable, lodged in the spine, I had nothing to do but to use the catheter twice or three times a day.

Now, the *Master* performed an operation, the impression of which will last, it was daguerreotpyed, graphic. An incision had been made; he held in one hand a forceps, and the other hand disappeared. It had gone downwards as in a pocket, in search for the ball; as I heard and expected, without success.

These few cases may serve as samples of the author's "rich treats," belonging to his "surgical feast," (page 280). The very learned gentleman, disappointed in not having received the charge of the boat, felt badly, and more so, when he found out that "a Dutchman" was entrusted with that office.

But, *me Hercule!* There comes yet another Dutchman! "And when it was found out by the Med. Director that he was incapable of taking care of himself, even—he added insult to injury, by sending a Dutch assistant-surgeon to supervise him."

That cruel Medical Director!—And to whom was the insult and the injury directed? I should think, against myself, if the matter had been really so. The author seems to be very anxious to exhibit his great force in logic. But I will not do wrong to him; perhaps that meanwhile that man's other eyelid became edematous, and that this Dutch assistant-surgeon only came to squeeze also pus out of it!

The truth is this: when the Medical Director found out that Dr. Roskoten had been wounded in the Battle of Shiloh; that he had, notwithstanding his own sufferings, voluntarily attended to the wounded on board the Hiawatha for full six days; and when he further heard that the same Dr. was outworn, suffering by hemorrhage of the lungs and lameness, so that he was incapable of going ashore after the provisions and stores, he acted very kindly in sending a very respectable medical officer to assist him in attending to these matters. It was Dr. Stahl, of the 10th Illinois.

On Saturday noon, I took charge of the boat; it was dark when we took in the last patients, and some 60 cots, from another boat. The number of sick and wounded were about 260.

As a great number of sick and wounded remained in tents, partially on wet soil—as I was informed, I considered it my duty to receive as many of them as I possibly could. The only difficulty was to get them on board that night. It was very muddy, the hills steep, and the wounded on top of them, at some distance. The conference with surgeons present, declared the transportation and the exposure for the wounded too difficult and dangerous; but I expected to succeed *the next morning* in bringing the balance of patients on board, as Captain Carson also stated that his pilot refused to go by so dark a night, unless he be compelled to lie by;—as far as I recollect he named the bridge.

The banks of the Tennessee River swarmed, at that time, with guerrilla bands. Our yellow flag was no protection against such outlaws. There was nothing to attach us to Pittsburg Landing, a barren place. Outworn to death nearly, I needed the quiet and comfort of my home, but I could not leave that night. I had a far more valuable cargo on board than the homesick professor; there were 260 sick and wounded soldiers entrusted to my hands. Could I leave them to the mercy of such cruel bands? Did the Dr. expect me perhaps to pilot the boat? How many more qualifications does he ask of an army surgeon?

Early on the following morning, I had my nurses at work, the cots were displayed, and things commenced to look well. The boiler-deck, thoroughly cleaned, became the most pleasant place of the boat, it could have afforded room for more than a hundred patients; the weather was calm and pleasant.

While a messenger was sent ashore for more patients, Dr. Simons came on board, about 9 o'clock A.M., I observed at the first glance that he had been influenced from a certain side. He asked me, shortly, why the boat was not on her way. I felt not inclined for any explanations, only stated my intention to receive more wounded men. He said, I had enough. His time being over-taxed in other places, he found no opportunity for examining the capacity of our large boat. Judging him by the past, I feel certain that he never used such language as the author alleges him to have used. He is a gentleman.

The Dr. says, further: "He (Dr. Simons) gave me the order, I carried it to the Captain, and we were off."

Not exactly so, Sir! With my permission the boat stopped alongside another boat *for several hours*, in order to take in a number of articles provided for by Dr. Douglas,—when I gave "the order, and we were off."

Why does the author not enter the army? If he could accomplish so much good in about 60 hours, why does he withhold his inestimable services from the nation's sickbed? Why does he not go at once before the U. S. Army Board? There is no want of vacancies. Quite a number of surgeons have resigned, outworn and with broken down constitutions, others were killed or wounded.

But it would not pay! And if there would be no ice in winter, or heat and dust in summer. But the worst of all is, that the self-planted laurels seem not to flourish so luxuriantly in the modest field of the army surgeons.

In recapitulating the whole article, his professorship seems to be so perfectly saturated with, and conscious of, his dignity as to feel himself an authority. This must of course proceed from his connection with Rush Medical College. The symbol of the medical profession ought to changed; for Æsculap's serpent there ought to be substituted the snake of the Paradise, as the tree of knowledge seems, in the author's opinion at least, only to grow in that modern Eden, and mainly to root in the professor's chair. Teachers and pupils, from all other Universities, will have to come to him to be instructed in his "Ethics," and to learn wisdom.

There will be a pilgrimage to that sacred place; the lights of

Boston, New York, St. Louis, Edinburgh, and Paris will fade away before this star of the first magnitude, not to mention Berlin or Vienna, as there are but "Dutchmen" in the chair.

A glorious time is coming, indeed. To assist in the heralding of the professor's glory, I will present a few of his delicious fruits, deposited in the *Chicago Medical Journal:*

"The boat was under charge of Dr. Roskiton, of Gen. McArthur's Divison. He is a German, and it is with some reluctance that I express my surprise that such a man can obtain the position he occupies. It convinces me that favoritism forwards more than merit. So far as I could judge, and it is the universal opinion of my companions as well, that he did not possess a single qualification for the place he filled. He was unable to get his outfit of stores; had the patients lying in utter confusion; and the only operation I saw him perform, was trying to squeeze pus from an edematous eyelid. When it was found out by the Medical Director that he was incapable of taking care of himself even—he added insult to injury by sending a Dutch assistant-surgeon to supervise him."

"The next day, (Saturday,) found us with our complement of wounded, near three hundred. We managed to get part of our army stores on board, and so far as any one of us could see, were ready to go. But it seemed almost impossible to get away. I asked our boat-surgeon what was keeping us; why not start Saturday night? He said the pilot did not want to start at night, and besides he had not enough wounded; if he started with so few, charges would be preferred against him. At that time every available point was occupied, except on the hurricane and boiler decks."

"In that condition, we stayed from Saturday until Sunday noon, with our two Dutchmen spinning around each other, as if practicing a new edition of the Lancers, until Director Simons came aboard, and I asked, what in humanity's name we were waiting for?—why these men, wounded and suffering, might not as well be lying in the Hospital at Mound City as in that boat? He could give no answer for the delay, except the stupidity of our asinine Dutchmen. I wish I could letter their hats. I

would substitute U. A. S. for the ones I saw them sporting. He gave me the order, I carried it to the Captain, and we were off."

Not quite so generous as the Dr. I shall not, nor can I reciprocate his desire to letter hats; he wore the U. S. very conspicuously, while I only used a citizen's hat, without that distinction, *my cap being torn on the battle field.*

But there are different ways of lettering things; it requires a very hot iron to brand a mule's back, while author's write their own epithets. R. ROSKOTEN,

St. Charles Hotel, Brigade-Surgeon.

CAIRO, *June* 21, 1862.

